# The endohyphal microbiome: current progress and challenges for scaling down integrative multi-omic microbiome research

**DOI:** 10.1186/s40168-023-01634-7

**Published:** 2023-08-26

**Authors:** Julia M. Kelliher, Aaron J. Robinson, Reid Longley, Leah Y. D. Johnson, Buck T. Hanson, Demosthenes P. Morales, Guillaume Cailleau, Pilar Junier, Gregory Bonito, Patrick S. G. Chain

**Affiliations:** 1 Los Alamos National Laboratory, Los Alamos, NM USA; 2 University of Neuchâtel, Neuchâtel, Switzerland; 3 Michigan State University, East Lansing, MI USA

**Keywords:** Multi-omics, Integrative bioinformatics, Endohyphal microbiome, Endobacteria, Mycovirus

## Abstract

As microbiome research has progressed, it has become clear that most, if not all, eukaryotic organisms are hosts to microbiomes composed of prokaryotes, other eukaryotes, and viruses. Fungi have only recently been considered holobionts with their own microbiomes, as filamentous fungi have been found to harbor bacteria (including cyanobacteria), mycoviruses, other fungi, and whole algal cells within their hyphae. Constituents of this complex endohyphal microbiome have been interrogated using multi-omic approaches. However, a lack of tools, techniques, and standardization for integrative multi-omics for small-scale microbiomes (e.g., intracellular microbiomes) has limited progress towards investigating and understanding the total diversity of the endohyphal microbiome and its functional impacts on fungal hosts. Understanding microbiome impacts on fungal hosts will advance explorations of how “microbiomes within microbiomes” affect broader microbial community dynamics and ecological functions. Progress to date as well as ongoing challenges of performing integrative multi-omics on the endohyphal microbiome is discussed herein. Addressing the challenges associated with the sample extraction, sample preparation, multi-omic data generation, and multi-omic data analysis and integration will help advance current knowledge of the endohyphal microbiome and provide a road map for shrinking microbiome investigations to smaller scales.

Video Abstract

**Supplementary Information:**

The online version contains supplementary material available at 10.1186/s40168-023-01634-7.

## Background

Communities of microbes that exist in a particular environment, often referred to as microbiomes, have been universally observed across diverse ecosystems and biological niches including food products, soils, humans, and animals [1–3]. Research into the roles of microbiomes has revealed that they perform important ecological functions, through direct impacts on their biological hosts in the case of the human microbiome, and directly within their natural environment in the case of soil microbiomes [2, 4]. Microbiome research has rapidly expanded over the past decade, primarily due to advances in multi-omics and biotechnology, which have enabled investigations at various scales of physical size and complexity. Consequently, this has altered our understanding of microbiomes and the concept of a holobiont — the idea that an organism and the compendium of associated microbes should be considered as a singular entity [5, 6]. The term holobiont has been extensively used to describe humans, plants, and animals; however, progression within the field also led to the notion that microorganisms, often only considered constituents of larger microbiomes, can harbor their own microbiomes. The concept of “microbiomes within microbiomes” has steered the field towards considering the diversity and functional roles of smaller-scale microbiomes (e.g., intracellular scale or smaller physical scale, with lower symbiont biomass) and their impacts on host functioning as well as the dynamics of holobionts within larger and more complex microbial consortia [7].

**Fig. 1:**
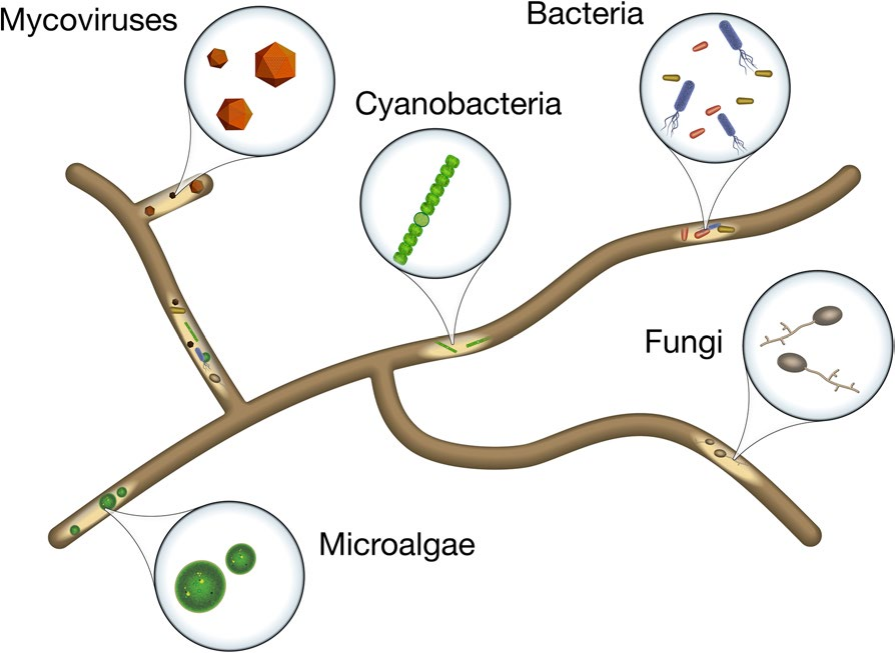
The known inhabitants of the endohyphal microbiome (microalgae, mycoviruses, bacteria [including cyanobacteria], and fungi)

Fungi are both common and integral members of environmental and host-associated microbiomes, and their presence and functional contributions are known to significantly affect microbiome dynamics and larger ecological processes [8, 9]. The biological complexity of fungi is often underestimated; however, fungi produce a number of complex structures as part of their life cycle, and they contain an array of organelles, lipid droplets, and other intracellular components. Furthermore, filamentous fungi are capable of harboring bacteria (including cyanobacteria), mycoviruses, and other fungi and can even internalize whole microalgal cells within their hyphae [10–14] (Fig. 1). As the diversity of observed endohyphal associates continues to expand, co-occurrences of these constituents have been more frequently observed, and it is becoming evident that many fungi harbor their own endohyphal microbiomes [15]. The endohyphal microbiome specifically refers to the microbiome found within living fungal hyphae and is the topic of this review. However, it is important to note that there are many known epihyphal associations where bacteria and other microorganisms live outside fungal hyphae in close physical proximity. More broadly, the mycosphere can be defined as the zone surrounding fungal mycelia that is characterized by elevated diversity and biological activity and is known to impact fungal physiology and ecosystem processes [16, 17]. As mentioned, this review will specifically discuss the endohyphal microbiome, but the ability to distinguish between epihyphal and endohyphal associates and their individual impacts on fungal host physiology and interactions in the mycosphere is an area of ongoing research in the field.

Multi-omics techniques have rapidly gained popularity for investigating individual constituents and smaller subsets of fungal-associating communities but have yet to be used to interrogate entire endohyphal microbiomes. Due to their early discovery relative to other members of the endohyphal microbiome, bacterial endosymbionts represent the most frequently studied group of fungal associates. In line with canonical microbiome function, endohyphal bacterial associates have been shown to significantly alter fungal host function, development, and interactions with other organisms [18–21]. Certain endohyphal bacterial associates have been well studied, and thousands of bacterial-fungal interaction pairs (some of which were shown to be endohyphal relationships) have been described [10]. Mycoviruses represent the second most widely studied group within the endohyphal microbiome, and currently, members of 23 viral families have been observed associating with hosts spanning the fungal tree of life [22]. While the total diversity contained within the endohyphal microbiome and its impact on fungal host biology remains unclear, further investigation into these areas will provide a more complete understanding of fungal contributions, interactions, and roles within microbiomes.

Multi-omics approaches (genomics, transcriptomics, proteomics, and metabolomics) have been foundational in elucidating the diversity and functional roles of the endohyphal microbiome. However, these investigations have also highlighted several challenges associated with performing integrative multi-omics studies on small-scale microbiomes. The development of methods and standards relating to sample preparation, data generation, data analysis, and data integration at these small physical (micro to nano) scales will be critical towards enabling holistic investigations of the endohyphal and other small-scale microbiomes.

Many recent microbiome studies using multi-omics methods have centered around scaling up and capturing snapshots of entire environments or ecosystems. Interrogating large-scale microbiomes using multi-omics is informative for analyzing high-abundance sequences and biomolecules but can lack the resolution required for capturing contributions of potential underlying microbiomes of the constituents. In order to interrogate smaller, but potentially equally biologically relevant microbiomes, current techniques and tools for larger-scale investigations must be adjusted, and the development of novel protocols and methods is also warranted. Herein, progress on the utilization of integrative multi-omics for endohyphal microbiome investigations will be discussed along with current and future challenges relating to the application of these techniques to holistic studies of small-scale microbiomes.

## Past and current usage of multi-omics for the endohyphal microbiome

**Fig. 2:**
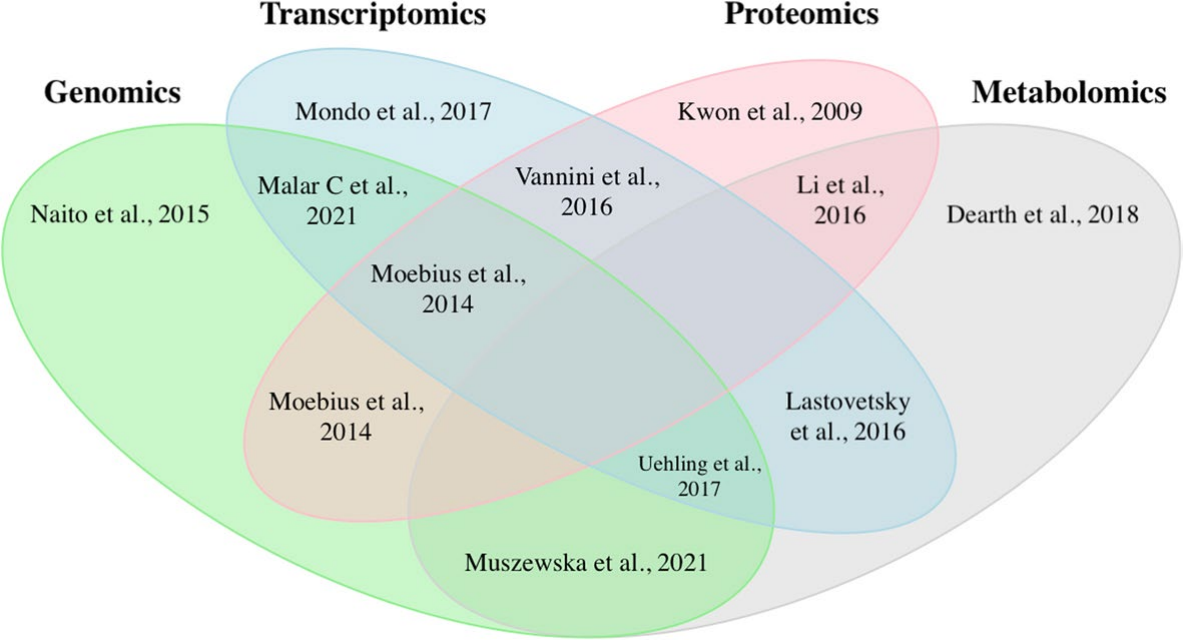
Combinations of omics techniques utilized in endohyphal microbiome studies. The publications listed in each Venn diagram section are not exhaustive: one example was chosen for each omics combination that had multiple reference options. The Moebius et al. (2014) publication is highlighted in two sections, and no publications have yet used the three remaining combinations of omics types (genomics + proteomics + metabolomics, transcriptomics + proteomics + metabolomics, and genomics + transcriptomics + proteomics + metabolomics) to investigate the endohyphal microbiome

Multi-omics investigations have provided key insights regarding the presence, taxonomic composition, and functional implications of members of the endohyphal microbiome on fungal hosts including impacts on host reproduction and pathogenesis [20, 23, 24] (Fig. 2). The publications portrayed in Fig. 2 can also be used as road maps for more specific methodological information for successfully conducting either individual or multi-omics-based investigations of members of the endohyphal microbiome.

### Endohyphal bacteria

Multi-omics investigations on endohyphal microbiomes have been most widely conducted on members of the Mucoromycota, which frequently harbor members from either one or both of two groups of highly studied bacterial endosymbionts: Mollicutes-related endobacteria (MRE) and *Burkholderia*-related endobacteria (BRE). Other fungal lineages, including Ascomycota and Basidiomycota, have been found to host endohyphal bacteria, but the distribution of these bacteria within fungal mycelial networks and their impacts on host physiology and functioning have been comparatively understudied [25–27].

Genomics- and metagenomics-based studies determined that MRE and BRE both have significantly reduced genome sizes and protein-coding content compared to closely related free-living bacteria, as well as reduced metabolic capacities [28–31]. These investigations have also revealed instances of horizontal gene transfer (HGT) between endobacteria and fungal hosts [29, 30]. Metagenomics has been utilized for simultaneous evolutionary and comparative examinations of both the bacterial endosymbiont and the fungal host, leading to insights into the origin of fungal-endobacteria interactions [28, 30, 32]. These genomic and metagenomic studies have provided fundamental knowledge around the endohyphal bacterial lifestyle and have demonstrated how these interactions can lead to genomic alterations of both the host and the endobacteria [33].

Transcriptomics has been used to uncover the genes and putative functions involved in interactions between endohyphal bacteria and their hosts. For example, comparative transcriptomic experiments between *Rhizopus microsporus* (Mucoromycota) isolates harboring BRE and those cured of their BRE partner revealed that the bacterial endosymbiont alters host gene expression, allowing it to control aspects of both asexual and sexual reproduction [34]. Comparative transcriptomic experiments have also provided insights into how a bacterial endosymbiont (*Luteibacter*, Gammaproteobacteria) interacts with its fungal host (*Pestalotiopsis*, Ascomycota) prior to endohyphal establishment [35]. Additionally, integrated analyses of transcriptome data with other omics data types have provided a more complete picture of the functional consequences of hosting endohyphal bacteria. For example, transcriptomic and proteomic data revealed that BRE in *Gigaspora margarita* (Mucoromycota) cause a shift in primary metabolism of the fungal host and an increase in fungal antioxidant production, which in turn alters interaction dynamics between plants and the fungal host [36]. Examination of the *R. microsporus* transcriptome and metabolome indicated differences in lipid metabolism and composition during interactions with its BRE partner [37]. To investigate the mechanisms by which bacteria can enter fungal host cells, genome mining, transcriptomics, and proteomics have been used to determine the critical role of chitinase during endosymbiosis establishment by *Mycetohabitans rhizoxinica* (Betaproteobacteria) in *R. microsporus* [38].

Metabolomics studies have identified broad shifts in the fungal metabolome related to harboring endobacteria. For example, the presence of BRE was found to induce substantial shifts in the metabolome of *G. margarita* [39]. Metabolomics has also been used in conjunction with genomics to determine that the presence of *Paenibacillus* (Bacilli) alters the lipid profiles of *Thamnidium elegans* (Mucoromycota) compared to other Mucoromycota fungi which do not host the bacterium [40]. Similarly, metabolomics was used in conjunction with genomics and transcriptomics in a study that determined that the presence/absence of a BRE (*Mycoavidus cysteinexigens*) substantially alters the *Linnemannia elongata* (Mucoromycota) transcriptome and metabolome, particularly with respect to fatty acid biosynthesis [28]. In addition to characterizing broad shifts in metabolomes, metabolomics has been used to identify important individual metabolites. For example, metabolomics was utilized to determine that rhizoxin, the causative toxin of rice seedling blight, is produced by a *Rhizopus*-associated BRE, not the fungus itself [41]. Proteomic and metabolomic investigations comparing spores of *G. margarita* with and without bacterial endosymbionts have shown that the endobacteria can significantly alter host protein expression and lipid profiles under several conditions and growth stages [42]. Investigations which integrated metabolomics and proteomics data identified key substrates exchanged between *Linnemannia elongata* (Mucoromycota) and its bacterial endosymbiont *Mycoavidus* sp. (Betaproteobacteria) [43]. Volatile emission measurements from *L. elongata* with and without *Mycoavidus* sp. showed that when the endobacterium is present, the production of fatty acids from the pyruvate pathway leads to high butyric acid and butyrate levels [44].

### Other members of the endohyphal microbiome

Exploratory studies involving other members of the endohyphal microbiome have lagged behind in numbers, but these studies have led to key findings. Multi-omic studies have facilitated the identification and corroboration of novel members of the endohyphal microbiome and have started to provide insights into how members within the microbiome interact with one another in addition to their interactions with the fungal host. Genomics and transcriptomics were used to explore genes of interest, potential HGT, gene expression, and phylogeny of the fungal host *Geosiphon pyriformis* (Mucoromycota), the first fungus found to internalize *Nostoc*, a nitrogen-fixing cyanobacterium [45]. Stable isotope labeling was used in the first ever study that observed the internalization of whole algal cells into fungal hyphae, which demonstrated a reciprocal exchange of nitrogen and carbon between the symbionts [14].

In addition to beneficial symbiotic partnerships, other endohyphal microbiome constituents such as viruses or parasitic fungi can modulate fungal host function. Transcriptomic analyses were used to identify the molecular changes of the mycoparasite *Ampelomyces quisqualis* (Ascomycota) during the colonization of its host *Podosphaera xanthii* (Ascomycota) [46]. Mycoviruses are typically single-stranded or double-stranded RNA viruses; therefore, RNA sequencing is needed to identify and classify most mycoviruses [12]. Recent surveys of publicly available fungal transcriptome sequencing data have revealed that mycoviruses are taxonomically diverse and are common among members of several fungal phyla [47, 48]. Transcriptome sequencing of *G. margarita* has also been used to investigate potential modulation of mycovirus diversity as a result of bacterial presence in the endohyphal microbiome [49]. Transcriptomics, proteomics, and metabolomics have been used to elucidate phenotypic and functional outcomes of mycovirus infections on fungal hosts [50–52].

While multi-omics techniques have advanced current knowledge on the diversity and impacts of the broader endohyphal microbiome, many of these studies focus on individual microbiome members. Due to the demonstrated utility of multi-omics and the growing recognition of the complexity of the endohyphal microbiome, we suggest the routine use of multi-omics in examining the full diversity of endohyphal microbiome composition and function over time. To initiate these more complex studies, it is necessary to identify and work to overcome the current limitations of these methods when applied to investigations of small-scale microbiomes.

## Challenges for integrative multi-omics of the endohyphal microbiome and other small-scale microbiomes

### Sample harvesting and extraction

The physical isolation of holobionts from their environments enables small-scale investigations without concerns of contributions from the larger microbiomes. The ability to isolate a fungal host in culture reduces or eliminates external contamination and unassociated genetic and molecular signals, permitting high-resolution investigations of the endohyphal microbiome [53]. However, this approach biases comprehensive multi-omic investigations into endohyphal microbiomes to only culturable fungi. Recent advances in laboratory devices and techniques have increased the efficiency of isolating fungi from larger microbial communities. Selective media, media additives, and growth conditions (e.g., incubation temperature) can be used to culture specific fungal groups [8]. Technical advances in single-cell and low-input biomass techniques have also enabled fungal investigations at smaller physical scales, allowing interrogation of hyphal fragments and micro-scale spores such as conidia.

When endohyphal microbiome constituents are separated from their fungal host and the rest of the microbiome, multi-omic analyses can be more straightforward and can result in higher quality data [54, 55]. However, it may be challenging or impossible to isolate or culture certain members of the endohyphal microbiome, as some associates may be unculturable and others are known to be dependent on the fungal host, such as the auxotrophic endohyphal bacterium *Mycoavidus cysteinexigens* which relies on its host for production of cysteine [28, 56]. Additionally, the process of separating these components leads to perturbations of the native sampling environment which can alter what is captured by transcriptomic, metabolomic, and proteomic approaches and lead to results that are not indicative of endohyphal functions. Therefore, separation of the microbiome components prior to enrichment or extraction may not always be preferable. It can also be difficult to axenically isolate the fungal host, as the success of methods for curing fungal hosts of their endohyphal bacteria remains highly variable. Current methods can be host or associate specific, and no standard methods exist for curing a fungal host of its entire endohyphal microbiome [26, 27]. This in turn limits comparative multi-omic experiments between cured and uncured fungal hosts to directly measure and assess impacts of the endohyphal microbiome.

For sequence-based interrogations, the nucleic acid extraction method can impact extraction efficiency of the endohyphal microbiome. This is partly due to differences in cell lysis requirements for host fungi and their microbiome constituents. Extraction kits for various microbiomes can differentially affect results, particularly in regards to nucleic acid recovery and quality [57, 58]. To identify and characterize mycoviruses present within the endohyphal microbiome, RNA extractions are often required. Total nucleic acid kits for simultaneous extraction of DNA and RNA have become more commonplace, especially for integrative multi-omics experiments. Although these dual-extraction kits can be very effective, reports have also documented biases with some of these extraction methods [59]. Various protocols exist for performing high-molecular-weight extractions on fungi for long-read sequencing, but not all of these protocols work efficiently for all lineages and cell types, and nucleic acid yields and quality can be highly variable [60, 61].

Metabolite and bottom-up protein extraction methods and purification efficiency can also vary for small-scale microbiomes. The selection of cellular disruption (e.g., physical or chemical) and metabolome or proteome extraction methods must be consistent with the platform used for data generation, and different methods can bias results or impact biomolecule recovery [62, 63]. Protein extraction methods can have variable impacts on protein yields and accuracy of protein identification; thus, it is important to evaluate which method may be most appropriate based on the experimental design and scientific goals [64]. Protein extractions from fungi can also be especially challenging given the robustness of fungal cell walls and fungal secretion of proteases which can cause protein degradation [65]. This becomes even more challenging when considering lysis requirements for both the fungal host and its associated microbiota. Metabolite extractions also rarely capture all metabolites in the sample, which can lead to loss in overall diversity or the need for multiple extraction methods [63]. Several published techniques, for example, the MPLEx (metabolite, protein, and lipid extraction) method, have pioneered the parallel extraction of proteins and metabolites from the same starting sample, thus making multi-omic experiments more integrative, less time-consuming, and more informative on a spatiotemporal scale for each particular sample [66–70].

**Fig. 3:**
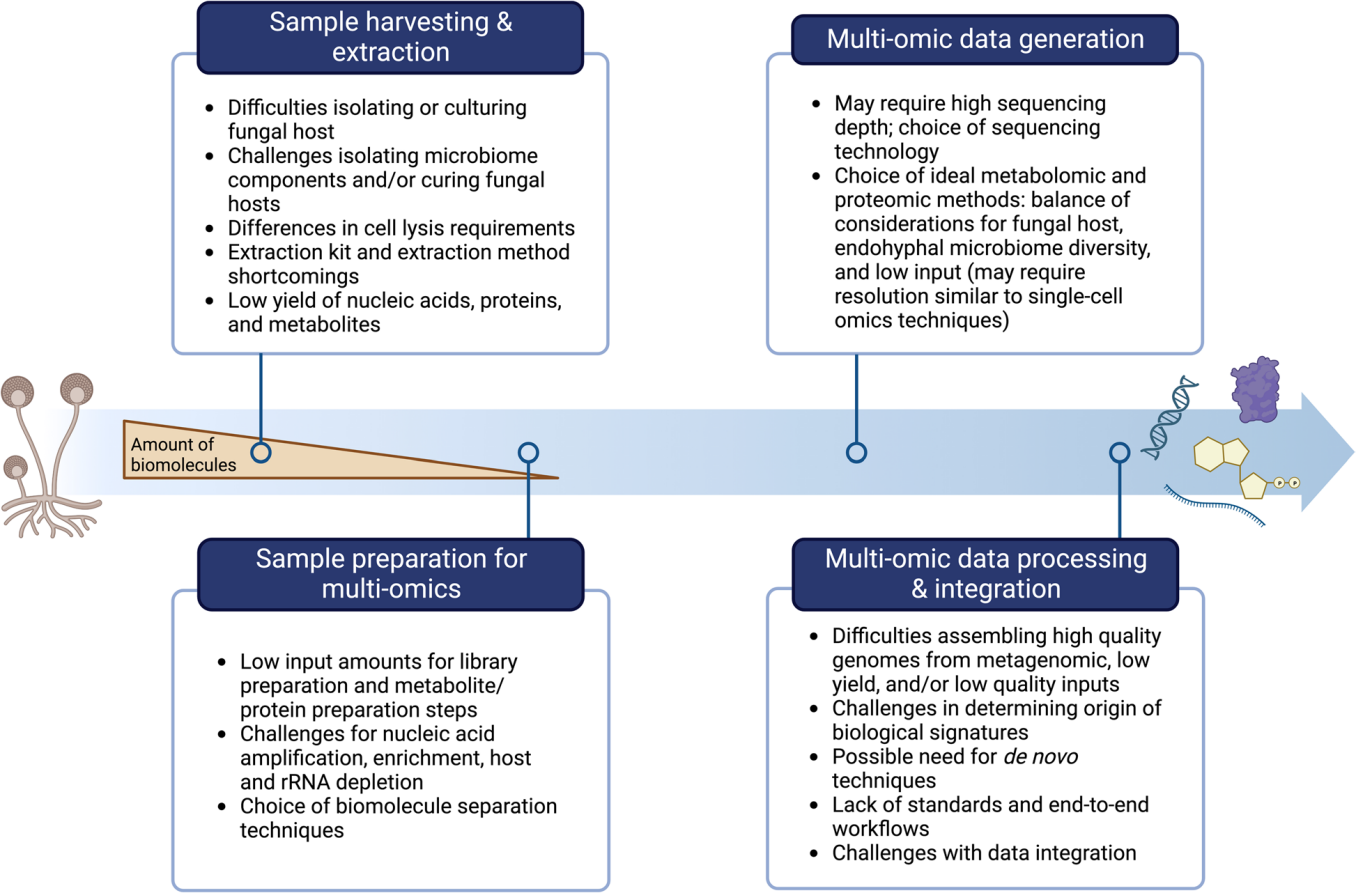
Challenges, considerations, and limitations for conducting integrative multi-omics experiments on the endohyphal microbiome. The overall multi-omics workflow consists of sample harvesting, sample extraction, and sample preparation, as well as data generation, data analysis, and data integration steps. The blue arrow denotes progression through the experimental process (from sample isolation to integration of multi-omics data), and the orange triangle denotes the decrease in sample yield as the workflow progresses from sample harvesting and extraction to sample preparation. Created with BioRender.com; adapted from “Multi-Panel Horizontal Timeline (Layout 2 × 2),” by BioRender.com (2023). Retrieved from https://app.biorender.com/biorender-templates

Sampling methods for extractions must also be considered when working with diverse fungal isolates. Filamentous fungi can vary substantially in their growth rates and hyphal division and production. All of these factors can impact the endohyphal microbiome, as microbiome constituents may replicate and disperse at different rates, creating differences in abundance across older and younger hyphae or hyphal segments. Certain members of the endohyphal microbiome may be present at very low levels, and not all members of the microbiome will have even distributions throughout the host’s hyphal network. Partial sampling of the fungal host, as opposed to collecting all available hyphae, could result in differential capture of microbiome components. Multiple extractions from total cultured fungal biomass or large portions of biomass at multiple locations across the hyphal network may be required to capture the diversity of heterogeneously distributed microbiome features. Spatiotemporal dynamics are important to consider as expression of transcripts, proteins, and metabolites will fluctuate on different time scales and differentially across individual hyphae. This can result in highly variable technical extraction replicates due to inconsistent capture of endohyphal constituent signatures. No extraction methods currently exist for the simultaneous extraction of DNA, RNA, proteins, and metabolites, making it important to consider splitting and preserving samples for each omic type. One of the biggest remaining challenges associated with performing multi-omics on the endohyphal microbiome is the consistently low yield of biomolecules produced by the microbiome constituents relative to the fungal host, which can be a significant hurdle for downstream multi-omics sample processing if the target signal is simply insufficient to provide robust or significant results. Figure 3 summarizes the aforementioned challenges as well as those associated with each step of the multi-omics experimental and analytical process.

### Sample preparation for multi-omics

Some considerations for the endohyphal microbiome sample preparation stage are relevant for all omics types, such as the consistent challenge of low input of molecular targets from the microbiome, compared with the overwhelming molecular targets from the fungal host. For investigations of nucleic acids, this challenge can be addressed through sequence enrichment, host depletion, and/or target amplification. Amplification techniques are the most straightforward and cost-effective of these options, as many primer options exist that will generally target barcode sequences for identification and taxonomic classification of bacteria/cyanobacteria (16S rRNA gene), viruses (RdRp, RNA-dependent RNA polymerase), fungi (ITS, internal transcribed spacer), and algae (18S rRNA gene). The use of these somewhat “universal” primers also means there is no reliance on prior knowledge of the specific diversity of endohyphal microbiome constituents, and this type of analysis provides a general overview of the community profile of the endohyphal microbiome. However, this can require multiple sample preparations with multiple amplification steps which can introduce additional biases and noise, and the results will not provide any genomic information outside of these barcode sequences. Whole genome or whole transcriptome amplification (WGA or WTA) methods can also be used to amplify more than the standard amplicon barcode regions. While newer methods have minimized biases (e.g. GC content biases), chimera generation, and artifacts that can result from these protocols, these issues are still observed to occur and can significantly affect mixed community samples [71].

Alternatively, enrichment and/or depletion assays can be performed. Hybridization-based genomic enrichment strategies have been used in microbiome research to select for known sequences based on lineage-specific signatures from organisms of interest [72]. However, this strategy relies on prior knowledge of taxonomic and sequence diversity within the sample, as efficiency is driven by sequence similarity between the enrichment probes and sequences in the sample. The design of specific enrichment and host-depletion panels can be quite time consuming and cost-prohibitive, especially when trying to encompass the potentially extensive diversity of the endohyphal microbiome. Our current limited knowledge on the diversity within the endohyphal microbiome makes it challenging to design enrichment panels since non-targeted sequences will generally not be captured or sequenced.

For fungal transcriptome investigations, it is common to deplete rRNAs or to perform polyA selection, as total RNA extractions are dominated by fungal host rRNAs [73]. PolyA selection limits complete characterization of the microbiome as non-polyadenylated sequences from viral or prokaryotic members are discarded, and this method can be less efficient at reducing rRNA levels compared to direct rRNA depletion methods [74, 75]. Furthermore, rRNA, polyA, and other forms of depletion require large amounts of starting material, and they often result in significant losses of total RNA. Considering the low proportions of RNA from members of the endohyphal microbiome relative to the fungal host, the resulting RNA yields may be too low for downstream sample and library preparation and subsequent analysis [75]. If the sample also contains abundant endohyphal bacteria, bacterial rRNA will likely dominate the remaining transcript pool post-depletion. Bacterial rRNA can also be depleted but will once again result in an additional loss of total RNA yield [76]. Library preparation for genomic and transcriptomic sequencing has historically been severely hindered by low nucleic acid input amounts. Newer methods for single-cell sequencing and low-input library preparation have helped to alleviate this shortcoming; however, these methods are still prone to errors, technical noise, and biases and can have higher failure rates [77–79].

Similar to nucleic acids, proteins and metabolites produced by the fungal host will be much more prevalent than biomolecules from endohyphal microbiome constituents. Compared with nucleic acids, proteins and metabolites cannot be amplified in a similar manner, but differences in the physical and chemical properties, including charge, hydrophobicity, and molecular weight, of biomolecules of interest (e.g., lipids, pigments, organic acids) can be exploited for selective enrichment. Recent advances in enrichment for single-cell omics show promise for improving the detection of low abundance analytes from endohyphal microbiomes. Capillary electrophoresis (CE) is commonly used for low input metabolite and protein selection (for bottom-up proteomics) based on biomolecule size and charge [80, 81]. While these enrichment techniques can select for specific classes of biomolecules, they also reduce total biomolecule amounts. This is particularly concerning with proteomic sample preparation, as the steps involved often lead to significant losses of proteins and peptides, and this is further exacerbated with lower starting inputs [82]. Many low-input proteomics protocols involve minimizing the sample preparation steps to reduce the sample loss characterized in conventional proteomics methods, often during tube transfer [82–84].

### Multi-omic data generation

The generation of sequencing data from endohyphal microbiome samples has unique considerations depending on the input sample type and research goals. Given the challenges listed above, metagenomic sequencing of a total fungal culture extract will often require deep sequencing to adequately assess the total diversity of the endohyphal microbiome. Deep sequencing can be very cost prohibitive, and most of the sequenced reads will belong to the fungal host. Rare members of the endohyphal microbiome may be captured at very low levels or not at all. Microbiome-enriched and/or fungal host-depleted metagenomic samples will reduce this need for ultra-deep sequencing. However, eukaryotic members of the endohyphal microbiome such as algae and other fungi can have much larger genomes compared to other members of the microbiome and require sufficient sequencing depth to acquire adequate genomic coverage.

The use of either short- or long-read sequencing technologies is also important to consider. Library preparation kits for short-read sequencing platforms generally require less sample input amounts than long-read platforms. However, single-cell/low-input library kits and workflows currently exist for both platforms for DNA, while only short-read platforms offer similar options for RNA samples. Short-read platforms are more commonly used for microbiome analyses, as their higher average sequencing depths and per-sample read counts are often superior for capturing rare microbiome members [85]. However, the use of long-read sequencing platforms in microbiome research is becoming more common. While long-read sequencing platforms may not be as efficient at capturing rare members of the microbiome, longer contigs collected from this platform aid in the generation of more complete genome references [86]. When possible, a combination of both long- and short-read sequencing platforms is ideal for these sample types [85].

Selection of an instrument or technique for performing metabolomic and proteomic analyses comes with its own set of challenges, as this requires considering methods best suited for investigating fungal hosts, microbiomes, and/or low-input (e.g., single cell) samples. Untargeted methods for both metabolomics and proteomics capture the most analyte diversity and capture unknown or undefined biomolecules produced by the fungal holobiont [87]. Techniques for performing untargeted low-input metabolomics and proteomics are still relatively nascent, but recent advancements have made this field more practical for small-scale microbiomes [88, 89]. The throughput of the technology must also be carefully considered (e.g., time per sample, amount of possible multiplexing), as rare analytes across samples may not be seen with lower throughput methods, even if the resolution per sample is ideal [90]. Mass spectrometry (MS) technologies are comparatively widely used for proteomic and metabolomic studies involving samples with low volumes and low biomolecule abundances, and MS instruments are continually improving in sensitivity and mass resolution [91].

Matrix-assisted laser desorption/ionization MS (MALDI-MS), secondary ion MS (SIMS), and electrospray ionization MS (ESI–MS) are currently some of the most popular low-input or single-cell metabolite and protein analysis platforms [92]. MALDI-MS has been widely used for single-cell metabolomics and proteomics, and it has shown promise for detecting analytes expressed from rare cells within a larger population [93]. However, MALDI-MS requires extensive sample preparation steps that may lead to additional sample loss and may compromise the natural metabolic states of the endohyphal microbiome constituents, and the technique does not perform well with low-molecular-weight organic acids [89]. SIMS provides one of the best possible cellular resolution ranges of currently available instrumentation, making it ideal for low input samples. However, it is still limited on mass resolution and can be a lower throughput technique. ESI–MS, especially when coupled with capillary electrophoresis (CE-MS), is also often used to perform low-input and small-scale metabolomics, even to the subcellular level [94, 95].

Omics techniques that also incorporate an imaging aspect can be critical contributions to investigations of microbiomes, including the endohyphal microbiome. Importantly, microscopy techniques are some of the best methods for distinguishing between members of the endohyphal and epihyphal communities. Imaging techniques also provide insights regarding the spatial heterogeneity within the fungal host and can be utilized to understand where biomolecules originate and end up within the fungal holobiont. Probes for fluorescence in situ hybridization (FISH) are traditionally designed from genomic and transcriptomic sequencing data. FISH imaging has been widely utilized for localization of endohyphal bacteria, and the technique could be expanded for the simultaneous detection and visualization of multiple members of the endohyphal microbiome [96, 97]. Single molecule FISH (smFISH) has also become much more reliable and applicable to rare microbiome constituents, and new methods have also increased the throughput of this approach [98]. Challenges and methods for visualizing endobacteria inside fungal hyphae using FISH have been previously discussed [97, 99]. Transmission electron microscopy has been used to identify MRE and BRE among other structures in the cytoplasm of Mortierellaceae and Glomeromycotina taxa to confirm their presence within fungal hyphae. Electron microscopy, in particular CryoEM techniques, continue to be invaluable for investigating the endohyphal microbiome [100, 101].

MALDI-MS can include an imaging aspect, which has even been used for 3D imaging within these small physical scales [102]. Recently developed metabolite imaging methods such as SpaceM directly correlate metabolomics data (e.g., MALDI data) with microscopy imaging to gain the spatial context of the cellular origin of the metabolites [103]. Various imaging techniques are difficult to integrate, as they each require unique sample preparation protocols (e.g., fixation); however, incorporating imaging methods into multi-omics studies will undoubtedly be indispensable for endohyphal microbiome studies. Imaging techniques also continue to be the most reliable way to validate which microbiome components are within the bounds of the fungal hyphae in order to define the endohyphal microbiome.

### Multi-omic data processing and integration

Obtaining genome sequences for members of the endohyphal microbiome is often the first step in multi-omics data analysis. These sequences serve as a reference for transcriptomics and proteomics and provide information on taxonomic diversity and functional potential, such as the presence or absence of metabolic pathways, within the endohyphal microbiome. If members of the endohyphal microbiome can be separated from the fungal host through filtration or centrifugation and sequenced individually, the genomic sequencing datasets from each individual can be analyzed using bioinformatic tools and/or pipelines tailored to their specific taxonomy (e.g., prokaryotes or eukaryotes). In cases where separation is not possible, metagenomic sequencing of endohyphal microbiomes will result in mixed datasets containing sequences from all members at variable coverage, with lower abundance constituents having the least amount of sequence coverage. Sequences from metagenomic datasets can be assembled, taxonomically classified, and assessed for quality using a number of established and taxa-specific approaches [104, 105]. Tools have recently been developed to selectively identify eukaryotic and viral contigs from shotgun metagenomics data to aid in the separation and subsequent analyses of certain contigs [106–108]. More specific tools have also shown promise for these analyses: the Spore-associated Symbiotic Microbes (SeSaMe) bioinformatics tool was specifically developed for the sequence classification of microbial associates of arbuscular mycorrhizal fungi from metagenomic sequencing data [109]. For endohyphal microbiome constituents that are not adequately captured in shotgun metagenomic assemblies, more sensitive read-based taxonomic classification analyses can be used to detect their presence; however, the lack of contigs limits the analyses of other omics types to de novo (without a reference) methods. While a few genome assembly software packages have been developed for low input and single-cell applications, substantial optimization hurdles remain [110, 111].

Genome assemblies can be especially difficult for endohyphal bacteria due to their aforementioned rapid rates of evolution, divergence from available bacterial references, and the possible presence of multiple closely related populations within a single fungal host [29, 32, 112]. HGT can also occur as a result of the intimate interactions between endobacteria and their fungal hosts; furthermore, the mycosphere and fungal mycelia have been shown to be a niche experiencing increased bacterial HGT [16, 29, 30]. This creates additional challenges when assembling multiple low-coverage genomes simultaneously, as HGT sequences may be incorrectly assembled, and determining the origin of HGT sequences may not be trivial. Traditional techniques to evaluate genome completeness and quality may also not be appropriate for endosymbiont genomes, for the reasons stated above.

Adequate genome references are a traditional cornerstone for integrative multi-omics. Incomplete or low-quality reference genome assemblies can make it challenging to utilize transcriptomic analysis software and methods that rely on reference genomes. Tools for de novo transcriptome assembly such as the *Trinity* software can make transcriptomic analysis without reference genomes more feasible [113]. *Trinity* has been successfully utilized for de novo assemblies of transcriptomes and metatranscriptomes containing signatures from multiple kingdoms [114–116]. Other tools and methods for separating host and symbiont reads in holobiont systems have been designed that minimize the chimeras that can typically arise from de novo transcriptome analyses [117]. Assemblers specifically created for metatranscriptome samples may also be the most appropriate for diverse endohyphal microbiomes [118]. Importantly, the application of de novo transcriptome assembly methods to the endohyphal microbiome relies on sequencing datasets containing sufficient coverage of transcripts from each microbiome constituent. In the case of the endohyphal microbiome, it is also important to consider that samples obtained from different locations across the hyphal network or different timepoints could vary in their microbiome composition and the resulting endohyphal transcriptome. Performing metagenomic and metatranscriptomic analyses on the exact same sample is ideal; otherwise, artifacts or discrepancies may arise. Given that mycoviruses are typically RNA viruses, their assembly and classification from metatranscriptome data will require specific viral workflows and databases that contain sequences from other mycoviruses.

Proteomic analyses also heavily rely on adequate host genome and microbiome metagenome reference data. When targeting microbial consortia, and particularly poorly explored ones such as the endohyphal microbiome, it is common practice to construct a protein reference database from annotated assemblies generated from the community’s metagenomic and occasionally metatranscriptomic data. As discussed in previous sections, metagenomic analyses will often not capture complete genomes for all members of the endohyphal microbiome. To ensure a more complete taxonomic and/or functional assignment of proteomic data, reference genomes from similar taxa to those in the microbiome can be used as a substitute, or recently developed de novo peptide prediction methods can be employed. The use of reference genomes of species similar to those found in the microbiome may not adequately represent the expressed proteins which can lead to incorrect or uninterpretable results, and this method can be especially problematic when assessing rapidly evolving bacterial endosymbionts whose proteins may share very little homology with otherwise closely related taxa. De novo methods for proteomic sequence data analysis may be required, although these are relatively new and lack standardization in the field [119, 120].

Analysis of untargeted metabolomics data can be completed without genomic or metagenomic references; however, unique challenges remain. These untargeted analyses rely on databases for accurate metabolite identification against libraries of known metabolites. However, these databases are known to be largely incomplete, thus leading to large proportions of untargeted metabolomics data having “unknown” classifications [121]. Many databases are highly limited on representation of metabolites from fungi, and studies involving fungi often require analyses which query a number of separate databases [122]. The use of multiple libraries or databases will likely be required to account for all the potential taxonomic diversity found within the endohyphal microbiome, and this can make it challenging to perform holistic analyses.

Multi-omics-based studies are highly complementary, as examinations of the metabolome and proteome indicate changes in function, but genome and transcriptome investigations help reveal the genes and functional potential responsible for these changes. The combination of multiple omics types, and integration of the data, provides clearer interpretations and conclusions regarding the functions within the endohyphal microbiome and their impacts on the fungal host. Omics techniques and data generation have advanced tremendously in the past decade but still vary in their sensitivity and limits of detection. The utilization of multiple omics types helps to overcome the deficiencies of any single technique, and this approach is often required to determine the impacts of the endohyphal microbiome on the fungal host, as altered expression of biomolecules within the endohyphal microbiome can directly impact gene expression or functions of the fungal host. The process of multi-omic data integration still lacks optimization and standardization, although several tools and workflows exist which have been previously discussed and compared [123, 124].

Research into small-scale microbiomes and microorganism holobionts will require additional efforts towards new and optimized algorithms, bioinformatic tools and workflows, and standardization specifically centered around analyses of low-yield interkingdom samples. There has been a very active campaign towards increased standardization in multi-omics bioinformatics workflows; however, significant shortcomings and gaps remain, particularly for microbial community analyses, making it difficult for many researchers to easily perform end-to-end multi-omic analyses [89, 125]. Currently, no standardized methods exist for holistically investigating the endohyphal microbiome using multi-omics, and standardization will be key towards progressing this field and making these insights comparable across different studies (e.g., comparing molecular underpinning of different fungal hosts and their respective microbiomes) and applicable to other small-scale holobionts.

## Conclusions

Many advances have been made in integrative multi-omic approaches for investigations into certain members of the endohyphal microbiome. However, significant challenges still remain at each experimental and analytical step that prevent fungal holobionts and other small-scale microbiomes from being routinely and holistically investigated. Integrative multi-omics is challenging even with abounding sample inputs into each omic analysis and with high-quality genome assemblies, and the challenges associated with integrative multi-omics are greatly compounded as additional members of the endohyphal microbiome are simultaneously interrogated and as additional omics types are integrated. The concept of a complex endohyphal microbiome is still relatively new, and the total taxonomic and functional diversity of the endohyphal microbiome has yet to be uncovered. Multi-omics has the potential to vastly expand these investigations, but it is imperative that the microbiome field considers the importance of these microbial holobionts and small-scale microbiomes when designing and advocating for new tools, methods, techniques, and standards. Understanding the functional contributions of each individual microbiome to their respective hosts as well as to larger microbial communities will unlock new opportunities and scientific questions in the field of microbiome research.

## Data Availability

Not applicable.

## Abbreviations

BRE: *Burkholderia*-related endobacteria
CE: Capillary electrophoresis
ESI: Electrospray ionization
FISH: Fluorescence in situ hybridization
HGT: Horizontal gene transfer
ITS: Internal transcribed spacer
MALDI: Matrix-assisted laser desorption/ionization
MRE: Mollicutes-related endobacteria
MS: Mass spectrometry
RdRp: RNA-dependent RNA polymerase
SIMS: Secondary ion mass spectrometry
smFISH: Single-molecule fluorescence in situ hybridization
WGA: Whole genome amplification
WTA: Whole transcriptome amplification
